# Primary surveys on molecular epidemiology of bovine viral diarrhea virus 1 infecting goats in Jiangsu province, China

**DOI:** 10.1186/s12917-016-0820-7

**Published:** 2016-09-05

**Authors:** Li Mao, Wenliang Li, Leilei Yang, Jianhui Wang, Suping Cheng, Yong Wei, Qiusheng Wang, Wenwen Zhang, Fei Hao, Yonglong Ding, Yinhua Sun, Jieyuan Jiang

**Affiliations:** 1 Institute of Veterinary Medicine, Jiangsu Academy of Agricultural Sciences; Key Laboratory of Veterinary Biological Engineering and Technology, Ministry of Agriculture, National Center for Engineering Research of Veterinary Bio-products, Nanjing, 210014 China; 2 Suining Animal Husbandry and Veterinary station, Suining, 221200 China; 3 Hai’an Animal Husbandry and Veterinary Station, Hai’an, 226600 China

**Keywords:** BVDV-1, Goats, Genotyping

## Abstract

**Background:**

Bovine viral diarrhea virus (BVDV) is a pathogen of domestic and wildlife animals worldwide and is associated with several diseases. In China, there are many reports about genotyping of BVDV strains originated from cattle and pigs, and some of them focused on the geographical distributions of BVDV. Currently, the goat industry in Jiangsu province of China is under going a rapid expansion. Most of these goat farms are backyard enterprises and in close proximity to pig and cattle farms. However, there was very limited information about BVDV infections in goats. The objective of this study was to assess the frequency of BVDV infections of goats, the relationship of these infections to clinical signs and determine what BVDV genotypes are circulating in Jiangsu province.

**Results:**

From 236 goat sera collected from six regions in Jiangsu province between 2011 and 2013, BVDV-1 was identified in 29 samples from the five regions by RT-PCR. The BVDV-1 infections occurred with/without clinical signs. Eight different BVDV-1 strains were identified from these positive samples based on the 5′-untranslated region (5′-UTR) sequences, and further clustered into four BVDV-1 subtypes on the phylogenetic analysis. Three were BVDV-1b, two BVDV-1m, two BVDV-1o, and one BVDV-1p, respectively.

**Conclusions:**

To our knowledge, this is the first report to investigate the occurrence of BVDV and the genotypes of BVDV infecting goats in China. The results indicated that BVDV-1 infections were indeed present and the viruses were with genetic variations in Chinese goat herds. The information would be very useful for prevention and control of BVDV-1 infections in China.

## Background

Bovine viral diarrhea viruses (BVDVs) are members of the genus *Pestivirus* (family *Flaviviridae*), and cause considerable economic losses in cattle industry worldwide [1–3]. Traditionally, two different BVDV species have been recognized as BVDV-1 and BVDV-2, and co-circulated in cattle, sheep, goats and pigs [4–7]. In recent years, several atypical bovine pestivirus strains named HoBi-like pestivirus were detected from contaminated fetal bovine sera and infected animals [8]. Phylogenetic analysis of these HoBi-like viruses showed a closer relationship to the recognized BVDV-1 and BVDV-2, and these viruses have been proposed as a new pestivirus species, BVDV-3 or HoBi-like pestivirus [8–11]. BVDV-1 and -2 infections are associated with several cattle diseases, including subclinical infections, immunosuppression, acute diarrhea, respiratory diseases, reproductive failures, and mucosal diseases in persistently infected calves [2]. Reproductive disorders caused by BVDVs appear with various clinical signs including infertility, abortion, stillbirth and persistently infected calves depending on the stage of pregnancy a cow is infected at [7, 12].

Genetic typing of the pestiviruses is based on different genomic regions, such as 5′-UTR, N^pro^, and E2 genes [13–15]. The 5′-UTR is the most frequently analyzed portion of the genome [16], and provides meaningful phylogenetic inferences as this region has the highest degree of sequence conservation and efficient amplification by RT-PCR [13, 17]. Based on the 5′-UTR sequences, BVDV-1 strains have further been divided into 21 subtypes (1a-1u) [13, 17–20], BVDV-2 into four subtypes (2a-2d) [21, 22], and BVDV-3 species has also been divided into two genotypes of Brazilian and Thai origin [10].

Investigating on the molecular epidemiology of BVDV can provide invaluable information about the diversity of viruses in a population and, in turn, inform control programs, drive vaccine development and determine likely infection sources. So far, epidemiologic surveys on BVDV infections have been carried out in cattle, pig and yak populations in several provinces of China [19, 23–26]. These findings highlighted that corresponding measures should be taken for control and prevention against BVDV infections.

Recently, the goat industry has developed increasingly in Jiangsu province, China. There were over five million goats reared in the province during 2013 according to the official statistics. However, there were different sizes and kinds of the goat farms because the farmers had various economic conditions, most of them were backyard reared with various sizes and numbers, and some goat flocks were adjoined to pigs and cattle. The risk of cross infection might occur between cattle, pigs and goats, so we investigated the BVDV infections in the goat herds and further determined the subtypes of BVDVs on the basis of 5′-UTR genomic region amplification and sequencing.

## Methods

### Sampling

A total of 236 goat serum samples in the laboratory were from 31 farms in six regions of Jiangsu province, China during 2011 to 2013. Of the samples examined in this study, 71 were from goats with clinical signs of diarrhea or abortion, while the remaining 165 samples from the animals with no overt clinical sign. No BVDV vaccines have been used for animal immunization in Jiangsu province yet.

### RNA extraction and RT-PCR detection

Total RNA was extracted from the serum samples using TRIzol Reagent (Invitrogen), and suspended in 20 μL of ultrapure water, following the manufacturer’s recommendations. RT-PCR was carried out in a 50 μL reaction mixture containing 1× RT-PCR buffer (TAKARA, Bio, Inc.), 20 pM of Panpesti generic primers targeting 5′-UTR, 2 U of one-step Enzyme Mix (TAKARA, Bio, Inc.) and 4 μL of RNA for the expected product sizes of 290 bp [27], the reaction was run in a thermocycler (Mjmini, BIO-RAD) according to the following program: reverse transcription at 50 °C for 30 min, denaturation at 95 °C for 5 min, 35 cycles at 94 °C for 30 s, 54 °C for 30 s and 72 °C for 45 s, and termination with a final extension of 10 min at 72 °C. The amplification products were electrophoresed in 2 % agarose gels. Part of the interesting N^pro^ genes were amplified as the same method and the primer sequences were as followed: N1F: ATGGAGTTGATTTCAAATGAACT; N504R: GCAGCTTGAAACCCATAGAG, the expected product was 504 bp.

### Sequencing

The expected bands were excised and recovered from the agarose gels using the Axygen gel extraction kit (Axygen, Hangzhou, China), and cloned into the pMD-18-T vectors (TAKARA, Bio, Inc.). The recombinant plasmids of the three positive bacteria were sequenced. The resultant gene sequences were compared with the sequences in the GenBank database using the Basic Local Alignment Search Tool (BLAST) and submitted to GenBank for confirmation of their identities.

### Phylogenetic analysis

Sequence data were assembled and analyzed using the DNAStar version 7.0 package (DNAStar Inc., USA). Multiple sequences were aligned with the corresponding regions of BVDV-1, BVDV-2 and atypical BVDV reference sequences retrieved from GenBank by the ClustalW program. Phylogenetic reconstructions for genotyping were compiled using the 224 bp fragment of the 5′-UTR region and 504 bp of N^pro^ gene, phylogenetic trees were constructed by MEGA version 4.1. The reliability of the neighbor-joining tree was estimated by boostrap analysis with 1000 replicates.

## Results

### Sample detection

RNA extraction was performed directly from sera in order to exclude possible pestivirus contamination during virus isolation in cell culture. The 5′-UTR fragments, with expected size of 289–291 bp were positive detected out in 78 of the 236 samples (32.6 %, Table 1). The amplified fragments were purified, cloned and sequenced. The pestiviruses were further identified and the prevalence of BVDV was 12.3 % (29/236) (Table 1). The remaining 48 pestiviruses were identified as border disease viruses (BDV) and reported before [28]. One pestivirus strain to be further testified was not included now. The 29 positive BVDV sera were from most regions, including Nanjing, Xuzhou, Nantong, Zhenjiang and Huai’an, and no BVDV was detected in Suqian (Table 1). Fifteen BVDV positive goats had different clinical signs as three from Zhenjiang (2012) and six from Nantong with diarrhea, two from Nanjing and two from Zhenjiang (2013) suffering with abortion, and two weak lambs from Huai’an, the remaining 14 were clinically health (Table 2).

**Table 1:** The prevalence of pestivirus in Jiangsu province

Year of collection	Origin	Samples	Host	BVDV Positive	BVDV prevalence (%)	BDV positive	BDV prevalence (%)
2011	Nanjing	3	goat	2	66.7	0	0
2012	Xuzhou	55	goat	6	10.9	1	1.8
2012	Suqian	59	goat	0	0	1	1.7
2012	Nantong	105	goat	14	13.3	46	43.8
2012	Zhenjiang	7	goat	3	42.9	0	0
2013	Huai’an	5	goat	2	40	0	0
2013	Zhenjiang	2	goat	2	100	0	0
Total		236		29	12.3	48	20.3

**Table 2:** Details of the BVDV positive samples identified

Year of collection	Strains	Origin	Clinical signs (Quantity)	Subtypes	Accession numbers
2011	JS12/02	Nanjing	Abortion (2)	1o	KP749794
2012	XZ5-9	Xuzhou	None (2)	1b	KP749800
	XZ5-8	Xuzhou	None (4)	1b	KP749795
2012	BH789	Zhenjiang	Diarrhea (3)	1m	KP749799
2012	HA6-5	Nantong	None (4)	1m	KP749798
	HA2-12	Nantong	Diarrhea (6)/None (4)	1o	KP749802
2013	XY-3	Huai’an	Weakness (2)	1p	KP749796
2013	JR1-2	Zhenjiang	Abortion (2)	1b	KP749797

### Sequence analysis

Based on the 5′-UTR sequences, 29 BVDV samples were classified as eight BVDV-1 strains (Table 2): the strains JS12/02 and JR1-2 were identified from two abortion goats, BH789 from animals with diarrhea, HA2-12 from six diarrhea and four healthy goats, XY-3 from two weakness kids, HA6-5 from four healthy goats, the strains XZ5-9 and XZ5-8 from healthy goats, respectively. The 5′-UTR sequences of these strains were deposited in GenBank under following accession number: KP749794- KP749800, KP749802 (Table 2), the interested N^pro^ sequences of HA2-12, JS12-02 and JR1-2 were as KX218370-218372.

The 5′-UTR sequence analysis of eight BVDV-1 strains as Table 3 showed 83.4–99.7 % homology with each other, and 83.1–88.9 % and 83.4–100 % identifies with the reference strains NADL (BVDV-1a) and VEDEVAC (BVDV-1b), respectively. The strains JR1-2, XZ5-8 and XZ5-9 from two regions (Zhenjiang and Xuzhou) shared very high identity of 100, 96.5 and 96.2 % with the BVDV vaccine strain VEDEVAC, respectively; BH789 (Zhenjiang) and HA6-5 (Nantong) showed a high identity of 95.5 and 94.8 % with the representative BVDV-1m strain ZM-95 originated from pigs in 5′-UTR, respectively. Compared with the reference strain IS25CP/01 (BVDV-1o), the strains JS12/02 (Nanjing) and HA2-12 (Nantong) shared nucleotide homology as low as 85.9 and 88.4 % homology respectively (Table 3). Based on the N^pro^ sequence, the strain JR1-2 was 98.2 % homology with the VEDEVAC (BVDV-1b), JS12/02 and HA2-12 had 97.6 and 98.2 % identity with the strain IS25CP/01, respectively.

**Table 3:** Identity of 5′-UTR between the new strains and reference strains

Reference strain	1b (VEDEVAC, 289 bp)			1m (ZM-95, 290 bp)		1o (IS25CP/01, 256 bp ^a^)		1p (BJ0701, 245 bp ^a^)
Strain	JR1-2	XZ5-8	XZ5-9	BH789	HA6-5	JS12/02	HA2-12	XY-3
Identity (%)	100	96.5	96.2	95.5	94.8	85.9	88.4	95.9

^a^The sequence information of reference strain was limited

### Phylogenetic analysis

The 5′-UTR phylogenetic tree was created using their nucleotide sequences and 44 BVDV reference strains covering all subtypes retrieved from GenBank, and the eight detected BVDV-1 strains were further subdivided into four subtypes (Fig. 2): BVDV-1b (XZ5-9, XZ5-8, and JR1-2), BVDV-1p (XY-3), BVDV-1m (BH789 and HA6-5) and BVDV-1o (JS12/02 and HA2-12), respectively. The same result showed in N^pro^ phylogenetic tree including the strains HA2-12, JS12-02 and JR1-2 (Fig. 3).

**Fig. 1:**
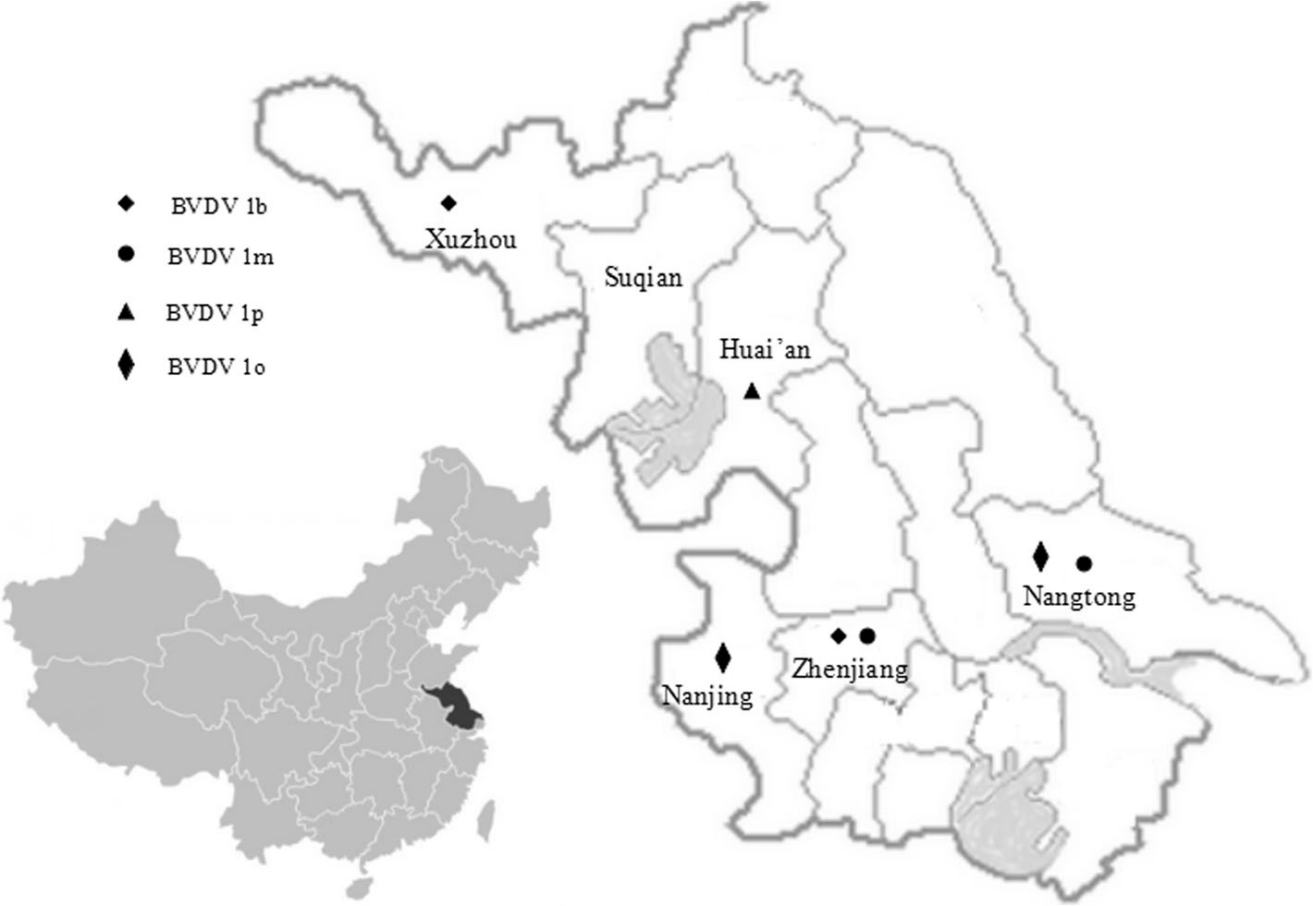
Geographical distribution of BVDV subtypes circulating in six regions of Jiangsu province

**Fig. 2:**
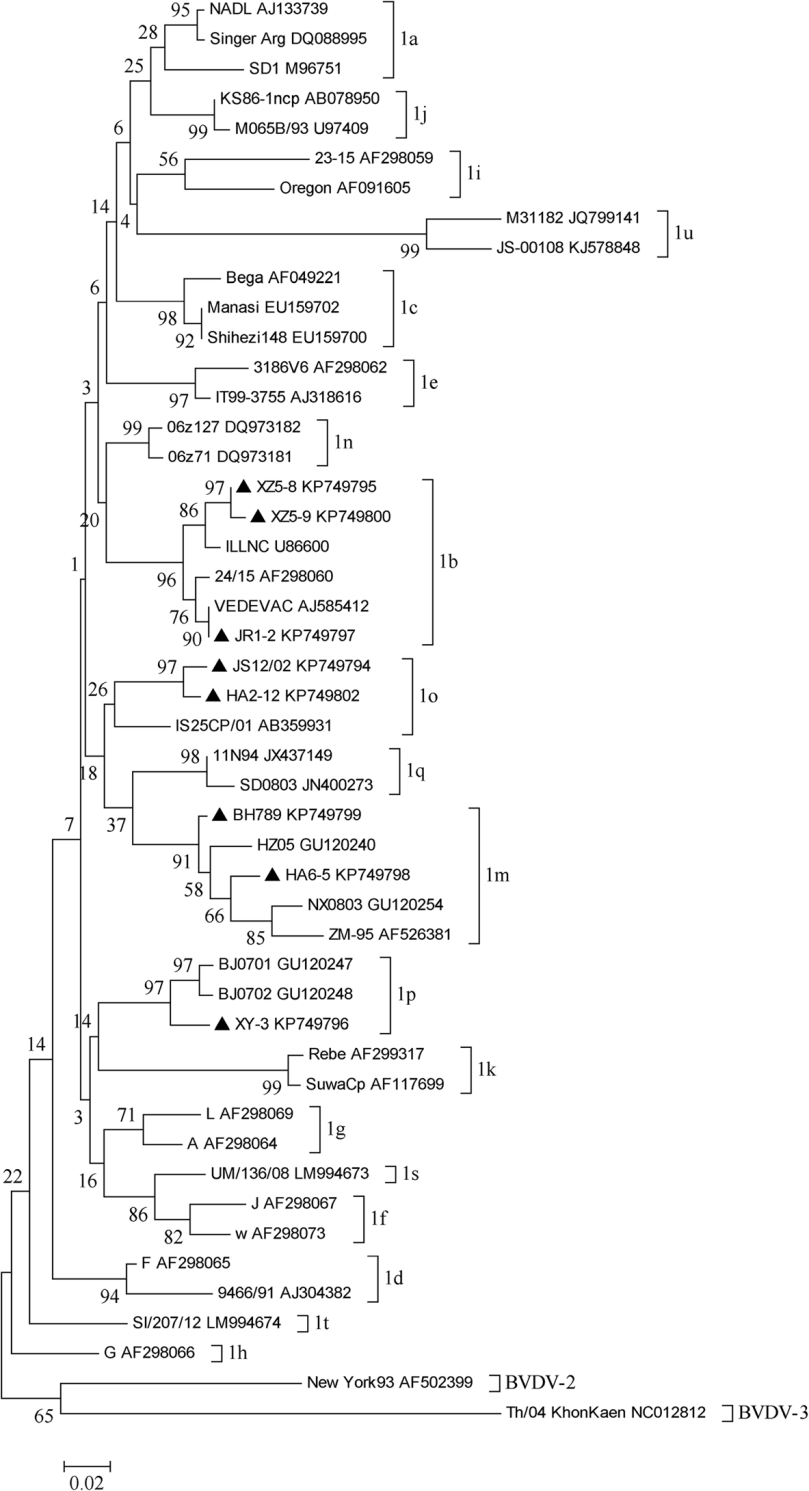
Unrooted phylogeneitc tree based on the 5′-UTR sequences. Phylogenetic tree of the 5′-UTR from eight BVDV positive samples was constructed by the neighbor-joining (NJ) method with the sequences published in GenBank. The nucleotide length of the 5′-UTR used for the analysis was 224 bp. The *numbers* at the phylogenetic branches indicated the bootstrap values (1000 replicates) in percentage supporting each group. The *bar* represented a genetic distance

BVDV-1b, BVDV-1m and BVDV-1o were detected in two regions, respectively; while BVDV-1p was only found from two weak goats in Huai’an (Table 2 and Figs. 1 and 2). Two strains XZ5-8 and XZ5-9 clustered into BVDV-1b were detected from Xuzhou. In Nantong, there were two circulating strains HA6-5 and HA2-12 that were classified into two different subtypes BVDV-1m and 1o; and two subtypes, BVDV-1m (BH789) and 1b (JR1-2) were detected from Zhenjiang as well (Figs. 1, 2 and Table 2).

## Discussion

Cattle are generally considered as the main host of BVDV infections, however, BVDV has also been found to infect an extensive range of animals including sheep, goats, swine, yaks, deer and members of the *Camelidae* family [4–7, 25, 29]. In China, BVDV infections were first detected in cattle in 1980 while the pig origin BVDV strain ZM-95 was isolated in 1995 [30]. BVDV infections in cattle, pigs, camels and Sika deer (Cervus nippon) have continually been reported in different regions of China [6, 18, 19, 23–26, 31–34]. The BVDV prevalence of pig population has significantly increased recent years [6, 26, 35]. Until now, the BVDV infections of goats in China were not examined, and the viral epidemic situation of BVDV in the goat flocks was not clear either. In this study, the serum samples from 31 goat herds of six regions in Jiangsu were detected and the results showed that BVDV infections were indeed present as 12.3 % of the prevalence in the samples. Eight different BVDV-1 strains were identified from five regions, and the infections might occur in the goat herds no matter the animals were with clinical signs or normal situations.

Currently, ten BVDV subtypes have been detected to be circulating among multiple hosts in China: BVDV-1a, BVDV-1b, BVDV-1c, BVDV-1d, BVDV-1m, BVDV-1o, BVDV-1p, BVDV-1q, BVDV-1u and BVDV-2a [18, 19, 23, 24, 26, 31, 33]. The four BVDV-1 subtypes determined in goats of Jiangsu were consistent with the previous subtypes reported from pigs and cattle on BVDV phylogenetic groups. At the same time, the different subtypes infecting goat herds might indicate that the epidemic situation of BVDV infections in the animal population of China was very complicated. The same subtype viruses found in cattle, pigs and goats meant the possibility of interspecies transmission of the viruses.

BVDV-1b was first isolated in Jilin province, China in 1980, and continually detected in Hebei, Xinjiang, Heilongjiang, Tianjin and Qinghai [23, 24], it is considered to be the major predominant subtype in Chinese cattle [23, 24]. The BVDV-1b strains JR1-2, XZ5-8 and XZ5-9 from two different regions were 96.2–99.7 % of identity with each other, and had very high identity (>96 %) with BVDV vaccine strain VEDEVAC [24], especially the JR1-2 had the 100 % identity in 5′-UTR and 98.2 % in N^pro^ gene sequences with the strain VEDEVAC, respectively, and these viruses also showed closed relationship based on the evolutionary trees. VEDEVAC was the main strain in batches of BVDV vaccine Oregon C24V in Hungary, however, no BVDV vaccines have been licensed for cattle and goats in China yet. Additionally, the current 1b strains shared 95.5–100.0 % identity with BVDV-1b isolates YL07 and TJ0802 originated from cattle in 5′-UTR gene, which showed that cross infections of the BVDV-1b viruses might occur between cattle and goats.

Chinese BVDV-1 strain ZM-95 of pig origin was first isolated in 1995 and classified into subtype BVDV-1m [30, 35]. There were no further reports until 2010, new BVDV-1m strains in Chinese cattle were detected [24]. According to the surveys, BVDV-1m infections appeared to be more popular in China recently [18]. As Table 3, two BVDV-1m strains BH789 and HA6-5 showed as high as 95.5 and 94.8 % identity with ZM-95, respectively. BVDV-1m infections in goats might be closely related with its prevalence in pigs since there were many pig farms in the regions. Some goats infected with BVDV-1m had diarrhea, however, the pathogenicity of BVDV-1m infections in goats was not clear yet.

**Fig. 3:**
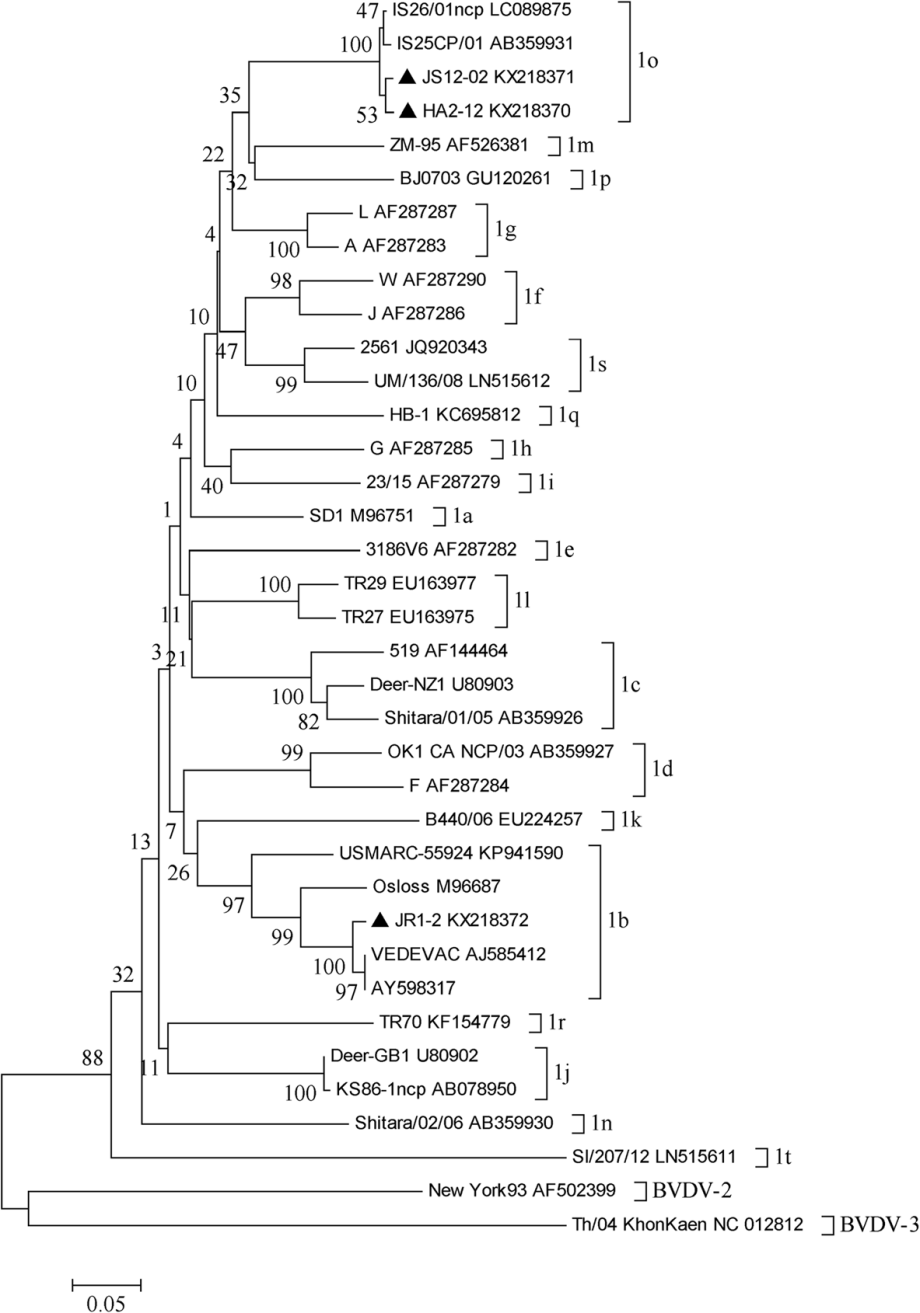
Unrooted phylogeneitc tree based on the N^pro^ sequences. Phylogenetic tree of the N^pro^ from three BVDV positive samples was constructed by the neighbor-joining (NJ) method with the sequences published in GenBank. The nucleotide length of the N^pro^ sequence used for the analysis was 504 bp. The *numbers* at the phylogenetic branches indicated the bootstrap values (1000 replicates) in percentage supporting each group. The *bar* represented a genetic distance

The BVDV strains JS12/02 and HA2-12 from goats were classified as BVDV1o, but they shared very low nucleotide identity (85.9 and 88.4 %) with the reference strain IS25CP/01 in 5′-UTR. The subtype viruses have been detected from bovine, pigs and camels in China [26, 32, 33], and the low prevalence of this subtype was observed worldwide. Interestingly, two BVDV-1o strains from goats shared high homology with each other, and significantly differed from other 1o strains based on and the Neighbor-joining unrooted trees of the 5′-UTR. Furthermore, these two viruses shared high homology (97.6 and 98.2 %) with IS25CP/01 strain based on the N^pro^ sequence. According to the phylogenetic trees (Figs. 2 and 3), the results showed that the present viruses might form one different branch with the 1o representative strains and the genetic divergence might exist within this subtype.

It should be noticed that four isolates detected from cattle in Beijing and Tianjing of China in 2010, formed a separate branch as BVDV-1p in the 5′-UTR phylogenetic tree, three of them were detected in the aborted bovine fetuses [24]. Now, the strain XY-3 detected from weak kids was in the same cluster with the BVDV-1p strains. It might indicate that the subtype viruses could infect animals to cause productive disorders.

This is the first report that BVDV-1 infections occurred in goats in Jiangsu province, China, and the genetic diversity of the viruses was determined as four BVDV-1 subtypes. Although numbers of the infected goats with clinical signs were limited, these infections might cause economic loss to the developing goat industry. More epidemiological investigations should be performed to better understand BVDV infections in goats as well as other farm animals, and design and develop effective control strategies.

## Conclusions

The study showed that BVDV were circulating in Jiangsu, and present as 12.3 % of the prevalence in the goats, eight different BVDV-1 strains were identified from the samples, which were further divided into four subtypes: 1b, 1p, 1m, 1o. BVDV cross infections might be occur between goats, cattle and pigs, for control and prevention against BVDV infections, epidemiologic surveys should be carried out in these animals.
